# Dysfunctional Social Support: Delivering Social Support at Work in an Unappreciative Way

**DOI:** 10.1007/s41542-024-00215-w

**Published:** 2025-01-22

**Authors:** Norbert K. Semmer, Wolfgang Kälin, Fabienne T. Amstad, Franziska Tschan, Nicola Jacobshagen, Terry A. Beehr, Mara Lehmann, Achim Elfering

**Affiliations:** 1 Department of Psychology, University of Bern, Fabrikstrasse 8, Bern, 3012 Switzerland; 2 University of Neuchâtel, Neuchâtel, Switzerland; 3 Central Michigan University, Mount Pleasant, MI USA; 4 Swiss National Centre of Competence in Research on “Affective Sciences”, University of Geneva, Geneva, Switzerland; 5 Pedagogical College Bern, Bern, Switzerland; 6 tumblebee, Oslo, Norway

**Keywords:** Social Support, Dysfunctional Support, Offense to Self, Strain, Attitudes

## Abstract

Self-esteem, both personal and social, constitutes a core concern for many people. Accordingly, Stress-as-Offense-to-Self theory focuses on threats, as well as boosts, to the self as important topics in occupational health science. Workplace social support is well established as a resource that signals acceptance and appreciation. At the same time, however, social support, notably support actually received, as opposed to perceived support availability, has been shown to have the potential to “backfire” and act as a stressor rather than a resource. The current study emphasizes the potential of social support to constitute a threat to the self if not delivered appropriately, that is, if it contains derogatory messages, even minor ones. The “*Bern  Dysfunctional Social Support Scale*” (*BDSSS*) focuses on such threats entailed in supportive attempts, focusing on provider behavior rather than recipient reactions and covering a broader range of (mostly subtle) derogatory behaviors than previously existing measures. In a cross-sectional study of 468 Swiss employees, it was associated with strain and attitudes in a way that characterizes it as a stressor. Effects were not strong, but dysfunctional support explained variance over and above demographic characteristics, neuroticism, classical social stressors, task stressors, and functional social support, as well as the outcome variables from a previous wave of measurement. The *BDSSS *therefore constitutes a valuable complement to existing measures. Although further research on this issue is needed, results underscore the need to sensitize employees and supervisors about pitfalls of support that is well intended but delivered in a potentially offending way.

## Dysfunctional Social Support: Delivering Social Support at Work in an Unappreciative Way

A positive self-evaluation can be regarded as an important personal resource (Hobfoll et al. 2018) and as a core concern for many people (Alicke & Sedikides, 2009); it is closely related to, and influenced by, a positive evaluation by relevant others (Lazarus, 1999; Leary & Baumeister, 2000; see also Kruglanski et al. 2022). The importance of a positive evaluation by self and others is underscored by phenomena such as self-serving attribution (Mezulis et al. 2004); hurt feelings when failure is attributed “to something that is an integral part of a person” (Hareli & Hess, 2008, p. 875), feedback avoidance in order to avoid threat to one’s ego or one’s image (Ashford et al. 2003), or the tendency to undermine others’ performance to preserve one’s self-esteem (Tesser, 1988), to name just a few (see Sedikides & Alicke, 2019; Tesser, 2001). Correspondingly, the threshold for detecting signs of (dis-)approval tends to be very low, and signals of (potential) threat are detected almost automatically (Leary & Baumeister, 2000). This low threshold implies that events or circumstances that constitute a threat, or boost, to the self need not be very strong and explicit to be attended to; rather, they may be triggered by rather subtle, and often implicit, cues. Studies on “subtly offending feedback” (Krings et al. 2015) or on the often rather mundane nature of behaviors experienced as appreciation (Stocker et al. 2014) demonstrate this low threshold.

Theory and research behind such results has inspired the development of the Stress-as-Offense-to-Self (SOS) theory (e.g., Semmer et al. 2019), which postulates that threats / boosts to the self are an important component of many stressful / resourceful experiences and therefore deserve to be emphasized more strongly in occupational health psychology. Given the importance of information about the self, SOS theory maintains that people tend to be vigilant to such information and therefore react to rather low-intensity cues; Semmer et al. (2019) use the analogy of a “’fire-alarm’ that is highly sensitive to signals of danger at any time” (p. 226).

The SOS-approach has led to the development of concepts in the domain of “identity-relevant stressors” (Thoits, 1991); examples are illegitimate tasks (e.g., Semmer et al. 2015), which are tasks assigned to employees that imply a disrespect for their occupational identity and therefore are considered a threat to the self, and “illegitimate stressors,” which reflect stressors caused by someone else who could have avoided creating the stressors by acting in a more considerate way (Kern et al. 2023; Semmer et al. 2021). On the resource side, research in the SOS context has focused on boosts to the self, such as experiences of success (Grebner et al. 2010), appreciation (Auer et al. 2024; Pfister et al. 2020; Stocker et al. 2014), and social support (Semmer et al. 2008).

Social support, the focus of the current study, constitutes a special case in this context: It typically constitutes a resource that is associated with positive effects (Bavik et al. 2020), but it also has the potential to “backfire” and become a stressor (Beehr et al. 2010; Deelstra et al. 2003; Gray et al. 2020; Nadler & Fisher, 1986). An important reason for such negative effects can be seen in the potential of social support to become a threat to the self (Fisher et al. 1982); the current paper introduces a scale assessing potentially threatening ways of delivering social support and analyzes associations of this “dysfunctional social support” with indicators of strain and well-being.

### Social Support

That social support can foster health and well-being is well established (Bavik et al. 2020; Cobb, 1976; Holt-Lunstad & Uchino, 2015; Mathieu et al. 2019; Viswesvaran et al. 1999). However, positive effects of social support are not always found (Beehr et al. 2010); negative effects, including moderator effects in terms of support augmenting, rather than reducing, strain, have been reported repeatedly (Mathieu et al. 2019). Negative effects typically are found with regard to support actually received (*received* support), as compared to support perceived to be available when needed (support *availability*). As support availability reflects “the expectation that others will provide support if needed” (Holt-Lunstad & Uchino, 2015, p. 186), helpfulness is implied; by contrast, specific support behaviors may not be as helpful as intended in the recipient’s eyes (Beehr et al. 2010; Deelstra et al. 2003; Gray et al. 2020; Lee et al. 2023).

#### Negative Effects of Social Support and the Role of Threats to the Self

Accounting for negative effects of social support, many authors refer to threats to the self; such threats, such as insults, may be rather explicit and direct (Beehr et al. 2010; Deelstra et al. 2003; Fisher et al. 1982; Gray et al. 2020; Hughes et al. 2022; Nadler & Fisher, 1986) or they may be implied indirectly, violating basic concerns that are close to people’s self and identity, such as unwanted (Floyd & Ray, 2017) or imposed support (Deelstra et al. 2003), which threaten the recipient’s autonomy.

Not all mechanisms regarding negative effects of social support are related to threat to the self. For one, support may create an undue focus on the negative aspects of the situation, which seems to “fuel” negative appraisal and emotions (Beehr et al. 2010; Fenlason & Beehr, 1994; Zellars & Perrewé, 2001). A second concerns suggestions that do not necessarily imply an offending message to the recipient but rather are inappropriate with regard to the problem at hand, such as doing things wrong as an example of “undependable social support” (Gray et al. 2020), although such inadequate problem-solving efforts may well affect self-esteem in the long run, as noted by Hughes and Gray (2024). Apart from these exceptions, however, the threat-to-self-esteem model can be regarded as “the leading explanation” for dysfunctional support (Gray et al. 2020, p. 363), which aligns well with the SOS model.

##### Behaviors Implying a Threat to the Self

There is a wide range of behaviors that may indicate threats to the self. Implied threats are not necessarily strong and explicit. Rather, as discussed above, the threshold for detecting potential threats is likely to be low, and people tend to perceive subtle nuances and to react to insinuations that are not expressed explicitly (Leary & Baumeister, 2000). This low threshold is a core tenet of SOS theory (Semmer et al. 2019); many examples for this tendency can be found in the literature on social support.

For instance, “*invisible support*” is considered especially helpful because it is not associated with feeling socially evaluated (Kirsch & Lehman, 2015) or incompetent (Bolger & Amarel, 2007), whereas “explicit help calls attention to recipients’ difficulties and undermines their competence” (Zee & Bolger, 2019, p. 316). Thus, the mere fact that support conspicuously comes along *as support* may already induce feelings of being regarded as incompetent. Furthermore, some negative effects of social support refer not to explicitly threatening elements but rather to the *lack* of positive elements that are considered essential for support to be effective. Thus, “*nonempowering*” support, that is, support that fails to foster self-reliance, “may not send the same signals about value and worth as receiving empowering help” (Lee et al. 2023, p. 774). The *responsiveness* concept regards making respondents feel “understood, validated, and cared for” as a necessary ingredient of effective support (Cutrona & Russell, 2017, p. 127), suggesting that a lack of responsiveness may suffice, and that no active denigration is necessary for support to be perceived as unhelpful. In line with this argument, research about the *timing* of advice in social support episodes shows that it makes a difference whether advice is offered early on or after a phase of acknowledgement and problem inquiry, implying that the lack of preceding acknowledgment and inquiry may render advice premature and undermine its value (Feng, 2009; Goldsmith, 2004; Pearlin & McCall, 1990). Summarizing research on support that is not helpful, Goldsmith (2004, p. 20) lists a number of rather inconspicuous aspects such as showing too little concern, minimizing or questioning the severity of the condition, or giving care without emotion, thus underscoring the capacity of rather subtle aspects of behavior to elicit negative responses. It might be noted that this aligns with research that identifies rather “low-level” behaviors such as “dismissive gestures” as examples of incivility (Bar-David, 2018) and with research demonstrating that subtle forms of discrimination have effects that are comparable to those of overt discrimination (Jones et al. 2016).

##### Assessing Support that Threatens the Self

Support considered unhelpful in general, and threatening in particular, has been manipulated experimentally (Deelstra et al. 2003); it also has been assessed by questionnaires (Beehr et al. 2010; Gray et al. 2020), which is also the method of assessment in the current study. Basically, there are two approaches, one asking about the reaction of the recipient (e.g., I feel incompetent), the other asking about the behavior of the support provider (e.g., insults me). Asking about the recipient’s reaction has the advantage of directly relating to the presumed process; it thus offers an indication that it is, indeed, the threat to the self that characterizes the reaction of the recipient. By contrast, describing behaviors has the advantage of offering indications regarding the nature of helpful versus unhelpful behavior. Furthermore, although the answers reflect the interpretation by the recipient, the more descriptive nature of behavioral items may make them less prone to biased responses due to personal characteristics of the respondent, such as neuroticism.

There are two questionnaire studies that have attracted attention in recent research on unhelpful support: Gray et al. (2020) and Beehr et al. (2010). The scale by Gray et al. (2020, p. 385) contains items referring to behavior, such as “criticize me while trying to help me tackle work problems;” “provide unwanted guidance when I don’t ask for it,” as well as items referring to reactions, for example “are not helpful when trying to comfort me.” Additionally, the scale contains items that do not focus on the message about the recipient but rather on appropriateness with regard to the problem, such as “do things wrong when completing a work task for me”. The items that refer to threats to the self in Beehr et al. (2010, p. 49) assess respondent reactions (feeling incompetent); in addition, Beehr et al. (2010) ask about unwanted support in behavioral terms (“… even if I ask him or her not to”).

Given the prominence of threats to the self and the importance of subtle behaviors implying such threats, a scale asking about a range of behaviors, including low-threshold behaviors, can yield important information about this kind of unhelpful support, its prevalence, and its effects; to the extent such effects can be shown, such a scale would also carry important practical implications that can inform interventions.

The * Bern Dysfunctional Social Support Scale* (*BDSSS*) was developed to capture such behaviors characterizing support that is intended to be useful or “functional” (Beehr, 2014) but actually are dysfunctional in the sense discussed above. By focusing on a range of provider behaviors and including subtle messages, the *BDSSS* differs from the most comprehensive scale on this topic, the “*Unhelpful Workplace Social Support Scale*” (*UWSSS*, Gray et al. 2020); that scale combines questions regarding behavior and questions regarding reactions, and the questions regarding threatening behaviors are more narrowly focused and concern strong and explicit threats (e.g., “insult me when providing advice”). In contrast to the *UWSSS*, the *BDSSS* does not contain questions on solutions that are inadequate with regard to the problem, such as doing things wrong when completing a task for the recipient. Thus, compared to the previous two scales, the *BDSSS* focuses on behaviors only rather than reactions to those behaviors, taps a broader range of dysfunctional support behaviors, and includes milder or more subtle behavioral offenses that are likely to be rather common in the workplace.

#### The Current Research

Aim of the current research is to introduce the *Bern Dysfunctional Support Scale* and to assess the effects of dysfunctional support on strain. Potential threat embedded in support should render it dysfunctional support, and thus a stressor, which should not simply neutralize positive effects but actually induce negative effects in terms of increased strain, as well as more negative and less positive attitudes. Using both work-related and general measures, we assessed strain by work-related irritation (Mohr et al. 2006) and by somatic complaints (Mohr, 1986). Work-related attitudes were job satisfaction (Judge et al. 2017) and intention to quit (Semmer et al. 2014), whereas positive attitude towards life (Grob et al. 1991) represents a more general construct.

We controlled for gender, which often is related to strain (Viertiö et al. 2021) and for trait neuroticism, which indicates proneness to experiencing stress and stress effects (Luo et al. 2023). Additionally, we controlled for traditional (i.e., functional) social support to ensure that dysfunctional support does not simply represent a lack of support. We also controlled for classical social as well as task-related stressors to allow for conclusions regarding effects beyond established work stressors. Controlling for outcome variables at an earlier point of measurement attenuates some of the problems associated with a cross-sectional design.

- Hypothesis 1: Dysfunctional social support is positively related to strain in terms of (a) work-related irritation and (b) somatic complaints.
- Hypothesis 2: Dysfunctional social support is negatively related to attitudes in terms of (a) job satisfaction and (b) positive attitude towards life, but (c) is positively related to intention to quit.

Our study contributes to the literature by following the call from Gray et al. (2020) to further investigate unhelpful social support, which is well documented but not yet well understood (see Hughes & Gray, 2024). Furthermore, we examine the unique contribution of dysfunctional support beyond functional support but also beyond neuroticism and “classical” stressors (including other commonly studied social stressors). Finally, controlling for previous values of outcome variables ensures that our results are unlikely due to reverse causal effects.

## Method

Data stem from the Swiss project “Working Experiences and Quality of Life in Switzerland: Work, Stress, and Personality Development” (ÆQUAS; Kälin et al. 2000). Follow-up studies were conducted 1, 2, 4, and 10 years after initial data collection in 1997 (Kälin et al. 2014)^1^. The * Bern Dysfunctional Social Support Scale* (*BDSSS*; Semmer et al. 2006) was only included at Time 5 (T5) in 2007, and the data used in the present study were from this wave, except for outcome variables from T4, which served as controls. Results from *ÆQUAS * have been reported previously, but no paper, including those using T5 data (Igic et al. 2017; Kälin et al. 2014), covers dysfunctional social support.

### Sample and Procedure

At the first wave (T1) in 1997, participants were in their last year of vocational training as nurses, cooks, salespeople, bank clerks, or electronic technicians (Kälin et al. 2000). T1 data were collected in vocational schools; follow-up questionnaires were sent to respondents who consented to be included in follow-up studies. The initial sample size was *N* = 1,394. In wave five, the number of participants was *n* = 571, constituting 40.9% of the initial sample. For the present analyses, sample size is *N* = 468, representing participants who took part in both waves 4 and 5. Of these, 60.7% were female, mean age was 31.2 years (*SD* = 3.1), 75.7% were in a close relationship, and 39.7% had children. The percentage of participants from the German speaking part of Switzerland corresponds roughly to their percentage in the country (61.3%), French-speaking participants are somewhat overrepresented (38.7%). At T5, 70.6% still worked in their respective profession as nurses (22.9%), cooks (8.8%), salespeople (9.0%), bank clerks (16.7%), and electronic technicians (13.2%); 29.5% had changed their occupation and were self-employed or worked in a variety of occupations as employees, in employment programs, or in casual jobs. Participants worked on average with a full-time equivalent of 85.7% (*SD* = 23.2%), which corresponds to about 36 h per week.

Compared with dropouts, respondents were significantly older, and more often married, female, and living away from home; they reported fewer social stressors but more task-related stressors than the dropout group (Elfering et al. 2007).

### Measures

Except for the dysfunctional support measure, which we developed ourselves, we used established scales. However, given that the project as a whole tried to cover many aspects of the respondents’ working life, some scales had to be shortened to avoid the questionnaire getting overly long. Decisions about which items to leave out were based on psychometric considerations, such as item-total correlations; if that was not possible, because all items had good psychometric properties, we used our own judgement concerning the optimal appropriateness of items for our study.

#### Dysfunctional Social Support

The scale was developed in several steps. In a first step, items were developed in brainstorming-type meetings by several of the authors, who had broad experience in occupational health psychology, including interviews with participants from various organizations, and who had taught lectures, seminars, and workshops on these topics at the university as well as to people working outside the university. Furthermore, five critical incident interviews of about 30 min with three male and two female participants from various occupations and a pilot study with 217 psychology students were conducted; in both cases, participants were asked to describe social support episodes they experienced as well intended yet not helpful, unpleasant, inappropriate, offending, or negative^2^. Reviewing the answers, five subject-matter experts removed / combined items that were redundant. In the following, we deleted items referring to subjective reactions (e.g., feeling uncomfortable) rather than behaviors, as well as items referring to support that was unhelpful for reasons other than a potentially offending message to the recipient, such as poor advice (see Gray et al. 2020).

To obtain a scale that was not too long yet covered relevant aspects well, Lehmann (2009) compared three versions that had been proposed in master’s theses (Hierzer, 2006; Kunz, 2008)^3^ and demonstrated that an 8-item version had very good psychometric properties, representing a single factor and displaying high internal consistency. This final version of the scale was employed in the current study; it is displayed in the Appendix (Table 3). Note that the items refer to behavior by support providers (e.g., combining support with reproaches) from the perspective of the recipient rather than to reactions by the recipient (e.g., not helpful). The lead-in phrase “How often does it happen that people at work help you in a difficult situation, but …” is followed by statements such as “suggest a solution prematurely,” with answers ranging from 1 *(almost never)* to 7 *(almost always)*^4^.

In line with our reasoning concerning subtlety, many of the items do not reflect strongly and explicitly offending behaviors. Accompanying help with reproaches and supporting someone reluctantly are two items that can be clearly designated as offending. Expecting infinite thankfulness is more indirect, inducing a feeling of indebtedness, which threatens equity perceptions and may affect self-esteem (Fisher et al. 1982; Golden & Schoenleber, 2014). Not acting like it goes without saying makes support provision conspicuously visible and thus increases the risk of feeling incompetent (Zee & Bolger, 2019); suggesting premature solutions without exploring the situation of the respondent indicates a lack of acknowledgement of the difficulty of the problem (Feng, 2009); similarly, not taking the problem seriously implies that the stress appraisal is not validated, which is an important component of “responsive” social support (Cutrona & Russell, 2017); the same applies to not really listening, and quickly responding with one’s own problems disregards the respondents and their situation. Thus, most of the items reflect rather indirect ways of failing to convey the message of care, esteem, and appreciation that can be regarded as the core message of functional social support (Cobb, 1976; Semmer et al. 2008).

#### Functional Social Support

“Available” social support at work (labeled functional social support in the present study) was assessed by asking “how much can each of these people be relied on when things get tough at work?” from Caplan et al. (1975) [German version by Frese (1989)]. Participants answered this question three times, concerning support from (a) one’s supervisor, (b) one’s closest colleague, and (c) other colleagues. Answers ranged from 1 *(not at all)* to 5 *(very much)*; internal consistency for the three items was α = 0.69.

#### Task and Social Stressors

*Task stressors *were assessed using five scales from the *Instrument for Stress-Oriented Task Analysis* (*ISTA*; Irmer et al. 2019; Semmer et al. 1995); these were time pressure (4 items, e.g., “How often must you finish work later because of having too much to do?”), concentration demands (4 items, e.g., “How often must you retain information that is difficult to remember?”), work interruptions (4 items, e.g., “How often are you interrupted by colleagues during your work?”), uncertainty about tasks (4 items, e.g., “How often do you receive unclear instructions”), and performance constraints (4 items, e.g., having to work with inadequate devices). As in other studies (e.g., Frese, 1985; Igic et al. 2017; Kälin et al., 2000; Meier et al. 2008), these stressors were combined into a task-stressor index; its composite score reliability (Nunnally & Bernstein, 1994) was 0.89.

*Social stressors* were assessed using the 8 item Form A (of two parallel forms) of the instrument by Frese and Zapf (1987). A sample item is “With some colleagues one often has disputes;” answers ranged from 1 *(not at all true)* to 5 *(very true)*; Cronbach’s alpha was 0.83.

#### Strain

Regarding work-related strain, we assessed work *irritation* (Mohr et al. 2006), reflecting negative affect such as feeling annoyed or angry, and difficulties unwinding after work. Of 8 items, 6 were selected, which had good item-total correlations. Sample items are “I have difficulties relaxing after work,” and “I get irritated easily, although I don’t want this to happen;” answers ranged from 1 *(absolutely not true)* to 7 *(almost absolutely true)*; Cronbach’s alpha was 0.84 / 0.87. 

Regarding general strain symptoms, 13 items on *somatic complaints* (Mohr, 1986) covered complaints such as headaches, fatigue, or nervousness during the past 12 months; answers ranged from 1 *(practically never)* to 5 *(almost daily)*; Cronbach’s alpha was 0.80 / 0.82.

#### Attitudes

As with strain, we focused on both work-related and general attitudes. *Job satisfaction* was assessed with 8 items. Seven items (Oegerli, 1984) ask how people lately felt about their work (e.g., “I hope my work situation remains as good as it is now”); answers ranged from 1 *(never)* to 7 (*always*; see Baillod & Semmer, 1994). The eighth item was a Kunin (1955) faces scale, with answers ranging from 1 *(exceptionally dissatisfied)* to 7 *(exceptionally satisfied);* Cronbach’s alpha was 0.85 in both waves. *Intention to quit* was assessed using two items from Bluedorn (1982) with timeframes of six months and two years, respectively: “If it were up to you, what is the probability that you still work in the same company in six months (item 1) / two years (item 2)?;” answers ranged from 1 *(very small)* to 5 *(very large)*; reliability (Spearman-Brown coefficient) was 0.83.

General attitudes were assessed as *positive attitude towards life*, using 5 of the 8 items by Grob et al. (1991) with good item-total correlations (e.g., “My life seems meaningful”); answers ranged from 1 *(absolutely not true)* to 6 *(absolutely true)*; Cronbach’s alpha was 0.85.

#### Neuroticism

Trait neuroticism was assessed with the 6 bipolar items (e.g., robust versus vulnerable) by Schallberger and Venetz (1999) on the basis of the German version (Ostendorf, 1990) of the *NEO Personality Inventory* (Costa & McCrae, 1985). Participants indicated how well these items described them, with answers being *very* (1 and 6), *quite* (2 and 5) and *somewhat* (3 and 4); thus, 1 / 2 / 3 indicated that the first pole (in the example: robust) described them very well /quite well / somewhat well, and 4 / 5 / 6 indicated that the other pole (in the example: vulnerable) described them somewhat well / quite well / very well. Cronbach’s alpha was 0.81.

### Statistical Analyses

We used multiple regression analyses, separately for each outcome. We controlled for gender, language-region, neuroticism, and the outcome variable at the preceding measurement. We further controlled for task-related and social stressors as well as functional social support.

We employed confirmatory factor analysis to assure measurement invariance of the latent construct dysfunctional support across language regions (French and German), using the eight items of the *BDSSS* scale as indicators. An unconstrained model yielded a rather good fit: χ^2^(32) = 104.80, *p* < .001, CFI = 0.97, TLI = 0.93, RMSEA = 0.070, 90% CI [0.055, 0.085]; and so did the model with items restricted to equal loadings: χ^2^(39) = 106.82, *p* < .001, CFI = 0.97, TLI = 0.95, RMSEA = 0.061, 90% CI [0.047, 0.075], with no significant differences: Δχ^2^(7) = 2.02, *p* = .96. Therefore, we can assume equivalence between the language-regions.

## Results

### Correlations

Table 1 displays descriptive statistics, correlations, and reliabilities for the study variables at T5 and the outcomes from the previous wave of measurement (T4). All scales had adequate internal consistency reliabilities.

**Table 1:** *Means (M)*,* Standard deviations (SD)*,* Zero-Order correlations*,* and reliabilities for study variables*

Variables	M	SD	1	2	3	4	5	6	7	8
*Individual characteristics*										
1. Language^a)^	1.39	0.49	--							
2. Gender^b)^	1.39	0.49	0.13**	--						
3. Neuroticism	2.78	0.72	− 0.04	− 0.23***	(0.81)					
*Stressors*										
4. Task	3.00	0.49	0.01	− 0.09	0.04	(0.89)				
5. Social	1.88	0.65	− 0.04	0.06	0.17***	0.40***	(0.83)			
*Support*										
6. Functional	3.70	0.79	− 0.28***	− 0.00	− 0.16***	− 0.11*	− 0.30***	(0.69)		
7. Dysfunctional	2.27	0.92	− 0.15**	0.00	0.19***	0.23***	0.47***	− 0.18***	(0.92)	
*Strain*										
8. Work irritation T4	3.00	1.00	0.11*	− 0.08	0.26***	0.20***	0.21***	− 0.13**	0.18***	(0.84)
9. Work irritation T5	2.85	1.11	0.16***	0.00	0.33***	0.33**	0.36***	− 0.21***	0.28**	0.41***
10. Somatic complaints T4	1.92	0.55	0.00	− 0.26***	0.28***	0.11*	0.15**	− 0.06	0.11*	0.43***
11. Somatic complaints T5	1.94	0.58	0.07	− 0.23***	0.34***	0.15**	0.19***	− 0.16***	0.21***	0.33***
*Attitudes*										
12. Job satisfaction T4	5.14	1.04	− 0.16***	− 0.02	− 0.19***	− 0.11*	− 0.29***	0.19***	− 0.18***	− 0.28***
13. Job satisfaction T5	5.08	1.05	− 0.22**	− 0.04	− 0.20***	− 0.33***	− 0.58***	0.38***	− 0.35***	− 0.20***
14. Intention to quit T4	2.58	1.32	− 0.13**	0.04	0.03	0.00	0.11*	− 0.11*	0.09*	0.06
15. Intention to quit T5	2.06	1.16	− 0.03	− 0.00	0.11*	0.16**	0.34***	− 0.24***	0.18***	0.05
16. Positive attitude towards life T4	4.73	0.69	− 0.16***	− 0.02	− 0.31***	0.01	− 0.22***	0.19***	− 0.16***	− 0.24***
17. Positive attitude towards life T5	4.76	0.71	− 0.19***	0.00	− 0.44**	− 0.08	− 0.30***	0.31***	− 0.27***	− 0.17***

Variables	9	10	11	12	13	14	15	16	17	
*Individual characteristics*										
1. Language^a)^										
2. Gender^b)^										
3. Neuroticism										
*Stressors*										
4. Task										
5. Social										
*Support*										
6. Functional										
7. Dysfunctional										
*Strain*										
8. Work irritation T4										
9. Work irritation T5	(0.87)									
10. Somatic complaints T4	0.25***	(0.80)								
11. Somatic complaints T5	0.42***	0.61***	(0.82)							
*Attitudes*										
12. Job satisfaction T4	− 0.22***	− 0.24***	− 0.15***	(0.85)						
13. Job satisfaction T5	− 0.39***	− 0.13**	− 0.23***	0.38***	(0.85)					
14. Intention to quit T4	0.04	0.08	0.04	− 0.45***	− 0.11*	(0.83)				
15. Intention to quit T5	0.14**	0.10*	0.11*	− 0.22**	− 0.56***	0.21***	(0.83)			
16. Positive attitude towards life T4	− 0.23***	− 0.26***	− 0.23***	0.44***	0.31***	− 0.15***	− 0.28***	(0.85)		
17. Positive attitude towards life T5	− 0.34***	− 0.21***	− 0.33***	0.27***	0.45***	− 0.07	− 0.32***	0.52***	(0.85)	

*Note. *Cronbach’s alphas in parentheses in the diagonal (except for intention to quit, which is based on the Spearman-Brown coefficient, and task stressors, which is based on the composite score reliability (Nunnally & Bernstein, 1994). ^a)^ 1 = German, 2 = French; ^b)^ 1 = female, 2 = male; *N *= 417-468; *p < .05, **p < .01, ***p < .001 (two-tailed)

Consistent with expectations, dysfunctional social support and functional social support were related to strain and attitudes in the expected direction. Also as expected, and corresponding to Gray et al. (2020), the two support variables were negatively associated, although not very strongly (*r* = − .18, *p* < .001); clearly, they are not simply opposite poles of the same construct.

### Regression Analyses

We had hypothesized that dysfunctional support would be associated with higher irritation (H1a) and somatic complaints (H1b) as well as lower job satisfaction (H2a) and positive attitude towards life (H2b), and higher intention to quit (H2c). As shown in Table 2, controlling for language-region, gender, neuroticism, task stressors, social stressors, and functional social support as well as the respective outcome variable at the preceding wave of measurement, dysfunctional social support explained unique variance in all outcome variables except intention to quit. Thus, four of our five hypotheses were supported.

**Table 2:** *Regressing strain and attitudes on dysfunctional social support and control variables*

	Strain						Attitudes								
	Work-related			General			Work-related						General		
	Work irritation			Somatic complaints			Job satisfaction			Intention to quit			Positive attitude towards life		
	B	se_B_	β	B	se_B_	β	B	se_B_	β	B	se_B_	β	B	se_B_	β
Outcome at T4 Language^a)^ Gender^b)^ Neuroticism Task stressors Social stressors Functional social support Dysfunctional social support	0.30 0.33 0.15 0.34 0.42 0.28 − 0.01 0.14	0.05 0.10 0.09 0.07 0.10 0.09 0.07 0.06	0.27*** 0.15*** 0.07 0.22*** 0.19*** 0.16** − 0.01 0.11*	0.53 0.09 − 0.09 0.12 0.05 0.02 − 0.04 0.07	0.04 0.05 0.05 0.03 0.05 0.04 0.03 0.03	0.51*** 0.08 − 0.07 0.14*** 0.04 0.02 − 0.06 0.12**	0.15 − 0.40 0.01 − 0.10 − 0.25 − 0.57 0.23 − 0.13	0.04 0.09 0.08 0.06 0.09 0.08 0.06 0.05	0.15*** − 0.19*** 0.00 − 0.07 − 0.12** − 0.35*** 0.17*** − 0.11**	0.16 − 0.15 − 0.02 0.06 0.14 0.31 − 0.33 − 0.01	0.04 0.12 0.11 0.08 0.12 0.11 0.08 0.07	0.18*** − 0.06 − 0.01 0.03 0.06 0.17** − 0.22*** − 0.01	0.34 − 0.18 − 0.03 − 0.26 − 0.01 − 0.08 0.13 − 0.08	0.04 0.06 0.06 0.04 0.06 0.05 0.04 0.03	0.33*** − 0.13** − 0.02 − 0.27*** − 0.01 − 0.07 0.14** − 0.10*
	Δ R^2 c)^ = 0.01* R^2^ = 0.37; R^2^_adj._ = 0.36			Δ R^2 c)^ = 0.01** R^2^ = 0.42; R^2^_adj._ = 0.41			Δ R^2 c)^ = 0.01** R^2^ = 0.46; R^2^_adj._ = 0.45			Δ R^2 c)^ = 0.00 R^2^ = 0.17; R^2^_adj._ = 0.15			Δ R^2 c)^ = 0.01* R^2^ = 0.40; R^2^_adj._ = 0.39		

*Note. *^a)^ 1 = German, 2 = French; ^b)^ 1 = female, 2 = male; ^c)^ ΔR^2^ = R square change from the last step where dysfunctional social support is added. *N *= 406-411; *p < .05, **p < .01, ***p < .001 (two-tailed)

Regarding the control variables, task stressors were associated with work-related irritation and job satisfaction, but not intention to quit, nor the general outcomes (somatic complaints; positive attitude towards life). Social stressors were associated with all work-related but not the general outcomes. Functional social support was associated with all attitudes (job satisfaction; intention to quit; positive attitude towards life) but not strain.

### Additional Analyses

Following the suggestion of an anonymous reviewer we first tested in additional analyses if neuroticism, social stressors, functional social support, gender, and language-region would constitute moderators. Only a single interaction emerged, which indicated that dysfunctional support was associated with somatic complaints more strongly when functional support was lower. Overall, therefore, our data do not suggest that the effects of dysfunctional social support are subject to boundary conditions to any meaningful extent with regard to the variables included in our analyses.

Second, to cross-validate our results, we first ran a G*Power analysis (G*Power 3.1.9.7; Faul et al. 2009) to determine the appropriate sample size. We used the a priori analysis method by testing the “linear multiple regression: Fixed R^2^ increase” (effect size [Cohens’s f^2^] = 0.02; power = 0.80, α = 0.05) with seven predictors plus one. This resulted in a *N* of 395. Next, a confirmatory factor analysis with this sample yielded a reasonable fit: χ^2^(32) = 84.47, *p* < .001, CFI = 0.97, TLI = 0.94, RMSEA = 0.065, 90% CI [0.048, 0.082]. Last, we used multiple linear regression analyses with a bootstrap sample of 5,000. The results for the regression analyses replicated the effects of dysfunctional social support as compared to the total sample (see Table 5 in the Appendix).

## Discussion

Stress-as-Offense-to-Self theory maintains that threats to the self are a core element of many stressful experiences at work, and pertinent research has provided quite some evidence supporting this approach (Semmer et al. 2019). In line with Leary and Baumeister (2000), SOS-theory also maintains that “identity-relevant stressors” (Thoits, 1991) do not require high intensity and explicit threats but that derogatory messages are likely to be perceived, and resented, even if they are rather subtle and implicit. The SOS-concept and its implications guided our approach to studying negative effects of a phenomenon that normally constitutes a potent resource when delivered in an appropriate and responsive way: social support. Negative effects of social support have repeatedly been shown (Beehr et al. 2010; Deelstra et al. 2003; Fisher et al. 1982; Gray et al. 2020; Nadler & Fisher, 1986), and according to the “leading explanation” (Gray et al. 2020) the main reason for such negative effects is likely that the “supportive” behaviors imply a threat to the self. Our research follows that tradition, and the *Bern Dysfunctional Social Support Scale* focuses on this proposition. Our results demonstrate that this type of support constitutes a stressor that is associated with strain and attitudes, both work-related and general, confirming our hypotheses for four of our five outcome variables.

The *BDSSS* differs from the measures by Beehr et al. (2010) and Gray et al. (2020) in three important ways. First, in contrast to the *UWSS* (Gray et al. 2020), which includes inadequacy regarding the *problem* (e.g., unreasonable solutions), the *BDSSS* focuses on inadequacy regarding social messages about the *recipient* in terms of behavior that is likely to be perceived as derogatory and not sufficiently appreciative. With regard to these derogatory behaviors, the dysfunctional support scale is more comprehensive than the *UWSS*. Second, the *BDSSS* items are behavioral and do not refer to the recipient’s reactions to the support provided, such as “not helpful” (Gray et al. 2020; appendix) or “I feel incompetent” (Beehr et al. 2010, p. 49). Third, the *BDSSS* avoids very strong negative terms such as “insult,” using less strong terms (e.g., “reproach”), and including more indirect and subtle threats such as making suggestions that appear premature and unresponsive because they are not preceded by acknowledging the problem and inquiring about it (Borrell-Carrió et al. 2004; Feng, 2009; Goldsmith, 2004; Pearlin & McCall, 1990), or emphasizing one’s helpful role and thus making the support conspicuously visible (see Zee & Bolger, 2019). The importance of such subtle characteristics of threatening messages is emphasized by SOS theory (Semmer et al. 2019); it corresponds to findings concerning “subtly offending feedback,” which is not explicitly offending but may indirectly insinuate that the receiver is incompetent, for instance by dwelling on minor mistakes even while maintaining a friendly tone (Krings et al. 2015), and it corresponds to research on subtle, as compared to drastic, examples of incivility (Bar-David, 2018) and discrimination (Jones et al. 2016). On the positive side, research also shows that appreciation reported by employees often refers to comparatively small things such as somebody saying thank you, being trusted with an interesting task, and the like (Stocker et al. 2014). The focus on subtlety also suggests itself because dysfunctional support is likely to be well-intended (see Gray et al. 2020), which makes subtle, as compared to drastic, deviations of optimal behavior especially plausible, often reflecting a lack of skills rather than an intention to offend. Pragmatically, the *BDSSS* entails the advantage of being rather short, as it contains only 8 items.

We tested associations of the *Bern Dysfunctional Social Support Scale* (*BDSSS*) with strain and attitudes, both work-related (work-induced irritation; job satisfaction; intention to quit) and general (somatic complaints; positive attitude towards life) in a sample of young people ten years after completing their vocational training. We controlled for demographic variables (gender, language region) but also for personality (neuroticism), conditions at work (task stressors, classical social stressors, and functional social support), as well as for the outcome at the previous wave of measurement.

Dysfunctional support explained unique variance in four of the five outcome variables, the only exception being intention to quit. Thus, our predictions were largely supported. The effects are not very strong, given that the variance explained after the control variables is 1%. Thus, dysfunctional social support does not seem to be a “core stressor” in the working lives of our participants. Nevertheless, as demonstrated by Rosenthal (1990) and Abelson (1985), such effect sizes are not negligible. Furthermore, dysfunctional support explained additional variance over and above (a) stability of the outcome, (b) neuroticism (i.e., the most pertinent personality variable, c) other important stressors (task, social), and d) functional support. Controlling for these variables constitutes a rather strict test. Notably, controlling for previous outcome values is known to substantially reduce effects as compared to concurrent associations that are not controlled for autoregressive effects (Orth et al. 2024). Thus, an important disadvantage of a cross-sectional design, that is, the explanation of an association in terms of the presumed outcome explaining the presumed predictor, can be ruled out. This seems important, although it does not render the design longitudinal, and other disadvantages of cross-sectional designs, such as the lack of control for the correlation between “predictor” and “outcome” at a previous point in time, remain. Altogether, these results therefore suggest that potential negative effects of support containing offending messages should be taken seriously and deserve attention in further research.

We are not the first to demonstrate such effects (see the literature cited above, as well as Rynek et al. 2022, who used an adapted version of the *BDSSS* in Germany; see below). However, as noted by Gray et al. (2020), further research is warranted to better understand support that is not helpful and to develop practical solutions (e.g., Hughes et al. 2022). Furthermore, although the experimental study by Deelstra et al. (2003) allows for causal conclusions, the two field studies by Beehr et al. (2010) and Gray et al. (2020) are cross-sectional; this also applies to the studies reported by Hughes and Gray (2024), although in their Study 2, measures are separated in time to minimize mono-method bias. Although cross-sectional as well, the current study controls for previous values of the outcomes; thus, pre-existing levels of these outcomes are unlikely to explain the results. Our study therefore complements existing studies in an important way.

The full range of characteristics rendering social support stressful requires additional research. The current study represents but one major part in the complex puzzle; it strengthens the threat-to-self approach that has characterized much pertinent theorizing (Fisher et al. 1982; Semmer et al. 2019), and confirms that helpful instrumental support should convey a message of care, esteem, and appreciation (Cobb, 1976; Semmer et al. 2008). Our study also underscores that there is a fine line between social support as a resource that may reduce, or buffer, the effect of stressors (Miner et al. 2012) on the one hand, and dysfunctional support as a unique social stressor that may threaten the self, on the other hand, because such threats may be induced by rather subtle indications of insufficient appreciation (see Semmer et al. 2008).

### Strengths, Limitations and Future Research

A strength of our study is that the sample was rather large and covered different occupations. Furthermore, although the study is cross-sectional in that associations refer to variables assessed simultaneously, controlling outcome variables at an earlier wave of measurement makes it unlikely that results are due to presumed outcomes predicting dysfunctional support. Furthermore, we controlled for task as well as classical social stressors, for functional support, and for neuroticism; these variables share variance with dysfunctional support as well as the outcome variables with regard to substance but also with regard to assessment by self-report. Both these sources of shared variance are thus controlled, which makes it unlikely that our results are primarily due to other conditions at work or to common method variance, although an influence of unmeasured third variables cannot be ruled out.

Regarding limitations, even with previous outcomes controlled, the study is not longitudinal; furthermore, measures are self-reported; although controlling for other self-reported measures renders a strong common method bias unlikely, a potential self-report bias remains (Podsakoff et al. 2012). Furthermore, the study refers to a rather special population of young people in a single country; it should be noted, however, that Switzerland is a multilingual country with considerable cultural differences, and the population consists of people with vocational training, which entails a large proportion of a typical Swiss cohort (Stalder & Nägele, 2011) and is not as highly qualified in terms of education as the samples in many studies in occupational health psychology. Furthermore, the *BDSSS* has been employed in Germany as well: Rynek et al. (2022) adapted the scale to refer to the situation of leaders working part time and attributing dysfunctional support to their working part time; they found negative associations with role identification and job satisfaction, mediated by perceived feelings of exclusion. Given that our measure of job satisfaction has not been employed very often and is not well known in English-speaking countries, it is important to note that Rynek et al. (2022) also found associations between dysfunctional social support and job satisfaction using items from a well-established measure (i.e., the Minnesota satisfaction questionnaire; Weiss et al. 1967).

Studies are needed on outcome variables specific to self-threat, such as feeling incompetent or derogated, and developments over time need to be investigated. Furthermore, future studies should address further boundary conditions for effects of dysfunctional support. For instance, a good relationship with the support provider might buffer negative effects of dysfunctional support. It might be interesting to employ the “*Unhelpful Workplace Social Support Scale*” (*UWSSS*, Gray et al. 2020) together with the *BDSSS* to assess the relative importance of problem-related inadequacies contained in the *UWSSS*, such as poor solutions as compared to behaviors that send a derogatory message to the recipient, which are more broadly covered in the *BDSSS*.

### Practical Implications

Our effects are not strong enough to justify large-scale interventions focusing specifically on dysfunctional social support. However, interventions aiming at conditions at work that make people feel supported and appreciated likely contain elements referring to social support (e.g., the CREW intervention; Leiter et al. 2011), and within such elements the pitfalls of dysfunctional social support should be addressed. Furthermore, apart from specific intervention efforts, organizations and leaders trying to follow concepts such as respectful leadership (van Quaquebeke & Eckloff, 2010) or health-oriented leadership (Krick et al. 2022) are well advised to pay attention to social support, including dysfunctional support in the sense discussed in this paper as well as in the more general sense of unhelpful support discussed by Gray et al. (2020). Because providers of dysfunctional social support may not realize its dysfunctionality, they may be offended by the receiver’s irritated reaction. Thus, people should be made aware of the – often subtle – nature of potential dysfunctional aspects of the support they provide and encouraged to avoid these wherever possible. On the other hand, as the recipients’ offense reflects a subjective appraisal of the supportive message, recipients might be supported in questioning their appraisal and encouraged to recognize dysfunctional support as well-intended; furthermore, they might be encouraged to acknowledge the helpful intention while pointing out the offense.

Finally, it is important to also consider the stress of the support provider. Chariatte et al. (2023) found that participants reacted to their partner’s work stress by providing support, both emotional and instrumental. In addition, however, their own work stress was associated with more dysfunctional support, which the authors attribute to depleted resources of the support provider due to their own work stress; Bodenmann et al. (2015) report similar results for experimentally induced stress. That stress of the provider increases the risk for social support being delivered in a dysfunctional way also confirms the conclusion of research on stress at work in general (Ganster & Rosen, 2013) that organizations should try to establish conditions for their employees that do not imply too high levels of stress. Overall, providing functional, and avoiding dysfunctional, social support can be regarded as important elements of good conditions at work, which involves job design with regard to task and social aspects (Parker & Knight, 2024) as well as their interplay with regard to social messages entailed in job design, such as trust and esteem (Semmer et al. 2016).

## Figures and Tables

**Fig. 1:**
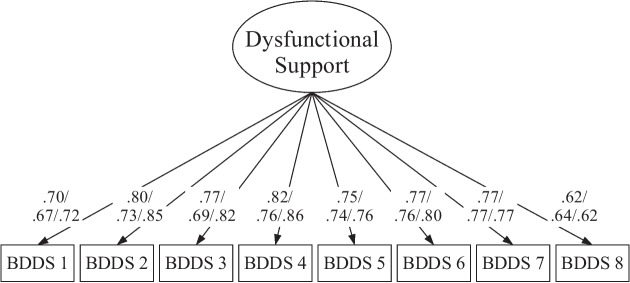
Results from the confirmatory factor analysis of the Bern Dysfunctional Social Support Scale (BDSSS) *Note*. *N*_*Total Sample*_ = 468: *N*_*Subsample 1*_ = 234, *N*_*Subsample 2*_ = 234; Standardized factor loadings (first line: total sample, second line: subsamples 1 and 2); Missing values are estimated using FIML (full information maximum likelihood) procedure; Fit statistics for the total sample: χ^2^(16) = 71.13, *p* < .001, CFI = .98, TLI = .95, RMSEA = .086, 90% CI [.066, .107]; for the subsamples (one analysis with subsamples 1 and 2 as subgroups): χ^2^(32) = 115.18, *p* < .001, CFI = .96, TLI = .92, RMSEA = .075, 90% CI [.060, .090]

## Appendix

Table 3, Table 4, Table 5 and Figure 1.

**Table 3:** *Means*,* Standard deviations*,* and Corrected item-total correlations of the* *Bern Dysfunctional Social Support Scale (BDSSS)*

“How often does it happen that people at work help you in a difficult situation, but …”	M	SD	*r*_it_
combine this with reproaches	2.02	1.04	0.67
support you reluctantly	2.05	1.03	0.78
expect infinite thankfulness	1.84	1.05	0.72
do not act like it goes without saying	2.06	1.09	0.76
suggest a solution prematurely	2.58	1.22	0.71
do not really take your problems seriously	2.26	1.18	0.77
do not really listen	2.49	1.24	0.76
quickly respond with their own problems	2.84	1.36	0.61

*Note.* *r*_*it*_ = Corrected item-total correlation

**Table 4:** *Results from the exploratory factor analysis of the Bern Dysfunctional Social Support Scale (BDSSS)*

Item	Factor loading		
	Total sample	Subsample 1	Subsample 2
combine this with reproaches	0.72	0.70	0.73
support you reluctantly	0.84	0.78	0.88
expect infinite thankfulness	0.76	0.67	0.81
do not act like it goes without saying	0.81	0.76	0.85
suggest a solution prematurely	0.74	0.72	0.76
do not really take your problems seriously	0.80	0.78	0.82
do not really listen	0.80	0.79	0.80
quickly respond with their own problems	0.63	0.64	0.62
Total variance explained	63.3%	59.2%	66.6%

*Note. N*_*Total sample*_ = 451-453, *N*_*Subsample 1*_ = 226-228, *N*_*Subsample 2*_ = 224-225; Subsamples 1 and 2 were randomly drawn (50% each) from the total sample. The extraction method was principal axis factoring with an oblique (direct oblimin) rotation. For all analyses, only one factor with an eigenvalue greater than 1 emerged. Therefore, factor loadings are based on the unrotated factor matrix. Further exploratory factor analyses with principal components factoring as extraction method and/or varimax rotation yielded comparable results

**Table 5:** *Regressing strain and attitudes on dysfunctional social support and control variables (random sample with bootstrapping)*

	Strain						Attitudes								
	Work-related			General			Work-related						General		
	Work irritation			Somatic complaints			Job satisfaction			Intention to quit			Positive attitude towards life		
	B	se_B_	CI 95%	B	se_B_	CI 95%	B	se_B_	CI 95%	B	se_B_	CI 95%	B	se_B_	CI 95%
Outcome at T4 Language ^a)^ Gender ^b)^ Neuroticism Task stressors Social stressors Functional social support Dysfunctional social support	0.32*** 0.33** 0.17 0.36*** 0.35** 0.33** − 0.01 0.17*	0.06 0.11 0.10 0.08 0.10 0.10 0.08 0.07	[0.21, 0.42] [0.12, 0.56] [-0.02, 0.36] [0.21, 0.51] [0.15, 0.55] [0.13, 0.52] [-0.16, 0.16] [0.04, 0.30]	0.50*** 0.09 − 0.08 0.11** 0.03 0.04 − 0.04 0.08*	0.05 0.05 0.05 0.04 0.06 0.05 0.04 0.03	[0.40, 0.61] [-0.02, 0.19] [-0.18, 0.02] [0.03, 0.19] [-0.09, 0.15] [-0.06, 0.13] [-0.12, 0.04] [0.02, 0.14]	0.14** − 0.37*** 0.01 − 0.11 − 0.13 − 0.61*** 0.24** − 0.15**	0.05 0.10 0.08 0.07 0.10 0.08 0.07 0.05	[0.05, 0.23] [-0.57, - 0.18] [-0.15, 0.17] [-0.25, 0.02] [-0.31, 0.08] [-0.76, - 0.44] [0.10, 0.39] [-0.25, - 0.04]	0.12* − 0.21 − 0.05 0.08 0.03 0.33** − 0.34*** − 0.01	0.05 0.14 0.12 0.09 0.12 0.12 0.09 0.08	[0.03, 0.22] [-0.48, 0.06] [-0.29, 0.19] [-0.10, 0.26] [-0.22, 0.27] [0.07, 0.57] [-0.53, - 0.17] [-0.17, 0.15]	0.33*** − 0.19** − 0.03 − 0.27*** 0.02 − 0.08 0.13* − 0.08*	0.05 0.07 0.06 0.05 0.06 0.07 0.05 0.04	[0.22, 0.43] [-0.31, - 0.05] [-0.15, 0.09] [-0.38, - 0.18] [-0.10, 0.15] [-0.22, 0.04] [0.02, 0.23] [-0.16, - 0.00]
	ΔR^2 c)^ = 0.01** R^2^ = 0.39; R^2^_adj._ = 0.38			ΔR^2 c)^ = 0.01** R^2^ = 0.38; R^2^_adj._ = 0.37			ΔR^2 c)^ = 0.01** R^2^ = 0.47; R^2^_adj._ = 0.46			ΔR^2 c)^ = 0.00 R^2^ = 0.15; R^2^_adj._ = 0.13			ΔR^2 c)^ = 0.01* R^2^ = 0.39; R^2^_adj._ = 0.38		

*Note. *^a)^ 1 = German, 2 = French; ^b)^ 1 = female, 2 = male; ^c)^ ΔR^2^ = R square change for the last step where dysfunctional social support is added. B, SE_B_, and CI 95% based on regression model with 5,000 bootstraps; R^2^, R^2^_adj._, and ΔR^2^ based on regression model without bootstraps. *N *= 347-352; **p *< .05, ***p *< .01, ****p *< .001 (two-tailed)

## Footnotes

1 At that time, no approval by an ethical board was necessary at our university for this type of questionnaire studies. However, we followed the guidelines of the Declaration of Helsinki and the Swiss Psychological Association, and the project conforms to the APA ethics policy regarding treatment of participants.

2 “Could you please describe a situation in which someone attempted to support you in a well-meant way, but you perceived this support as not helpful, disagreeable, or negative?”

3 We are indebted to Simone Hierzer and Melina Kunz for their contributions to the development of the final version of the *BDSSS*.

4 As recommended by an anonymous reviewer, we ran an exploratory factor analysis (principal axis factor analysis with oblique rotation); it yielded one factor with an eigenvalue greater than 1, which explained 63.3% of the variance. In addition, we ran (a) exploratory factor analyses and (b) a confirmatory factor analysis for two randomly drawn subsamples, which also resulted in a one-factor solution; these results are displayed in the Appendix (Table 4 and Figure 1).
